# Monitoring arthropods under the scope of LIFE-SNAILS project: II - Santa Maria Island data with implementation of the Index of Biotic Integrity

**DOI:** 10.3897/BDJ.14.e203118

**Published:** 2026-09-17

**Authors:** Martha Vounatsi, Sébastien Lhoumeau, Alexandra Dal Lago, Sophie Wallon, Nelson B. Moura, Mauro Ponte, Ricardo Abreu, Paulo A. V. Borges

**Affiliations:** 1 Azorean Biodiversity Group, School of Agricultural and Environmental Sciences, University of the Azores, Rua Capitão João d´Ávila, Pico da Urze, 9700-042, Angra do Heroismo, Azores, Portugal Azorean Biodiversity Group, School of Agricultural and Environmental Sciences, University of the Azores, Rua Capitão João d´Ávila, Pico da Urze, 9700-042 Angra do Heroismo, Azores Portugal; 2 University of Azores, CE3C—Centre for Ecology, Evolution and Environmental Changes, Azorean Biodiversity Group, CHANGE —Global Change and Sustainability Institute, School of Agricultural and Environmental Sciences, Rua Capitão João d’Ávila, Pico da Urze, 9700-042, Angra do Heroísmo, Azores, Portugal University of Azores, CE3C—Centre for Ecology, Evolution and Environmental Changes, Azorean Biodiversity Group, CHANGE —Global Change and Sustainability Institute, School of Agricultural and Environmental Sciences, Rua Capitão João d’Ávila, Pico da Urze, 9700-042 Angra do Heroísmo, Azores Portugal; 3 University of Bologna, Bologna, Italy University of Bologna Bologna Italy; 4 Secretaria Regional do Ambiente e Alterações Climáticas, Project LIFE SNAILS (LIFE20 NAT/PT/001377), Rua Dr. Teófilo Braga nº 10/12/14, 9580 – 535, Vila do Porto, Santa Maria, Azores, Portugal Secretaria Regional do Ambiente e Alterações Climáticas, Project LIFE SNAILS (LIFE20 NAT/PT/001377), Rua Dr. Teófilo Braga nº 10/12/14, 9580 – 535, Vila do Porto Santa Maria, Azores Portugal; 5 Secretaria Regional do Ambiente e Alterações Climáticas, Project LIFE SNAILS (LIFE20 NAT/PT/001377), Rua do Galo nº 118, 9700-040, Angra do Heroísmo, Terceira, Azores, Portugal Secretaria Regional do Ambiente e Alterações Climáticas, Project LIFE SNAILS (LIFE20 NAT/PT/001377), Rua do Galo nº 118, 9700-040 Angra do Heroísmo, Terceira, Azores Portugal; 6 IUCN SSC Atlantic Islands Invertebrate Specialist Group, Angra do Heroísmo, Azores, Portugal IUCN SSC Atlantic Islands Invertebrate Specialist Group Angra do Heroísmo, Azores Portugal; 7 IUCN SSC Monitoring Specialist Group, Angra do Heroísmo, Azores, Portugal IUCN SSC Monitoring Specialist Group Angra do Heroísmo, Azores Portugal

**Keywords:** Azores, Habitat quality, Long-term monitoring, Macaronesia, SLAM traps, Species records

## Abstract

**Background:**

The data presented here constitute an important component of the LIFE SNAILS project (Support and Naturalisation in Areas of Importance for Land Snails) which aims to protect three threatened endemic mollusc species from Santa Maria Island: two land snails (*Oxychilus agostinhoi* Martins 1981 and *Leptaxis minor* Backhuys, 1975) and a semi-slug, *Azorivitrina angulosa* (Morelet, 1860). Although, arthropods are not direct targets of the project, their communities can provide useful indicators of overall habitat quality. A previous survey of mixed forests on Santa Maria provided the baseline for the present study, as the same sites were surveyed, except two additional sites incorporated in 2025.

**New information:**

We obtained 55 sampling events from forests on Santa Maria between 16 June 2024 and 15 December 2025. A total of 20,282 specimens were collected, with 15,055 specimens identified to species or subspecies level, representing 74% of the total sample. Overall, 172 species and subspecies were identified: 27 endemic, 48 native non-endemic, 77 introduced and 20 of uncertain colonisation status. We recorded ten species new to Santa Maria Island. However, none represented a new record for the Azores Archipelago. Of these new island records, two were native non-endemic species, six were introduced and two had uncertain colonisation status. These new records include *Campyloneura virgula* (Herrich-Schaeffer, 1835), *Carpelimus troglodytes troglodytes* (Erichson, 1840), *Hermetia illucens* (Linnaeus, 1758), *Nacaeus impressicollis* (Motschulsky, 1858), *Neobisium maroccanum* Beier, 1930, *Phyllotreta procera* (Redtenbacher, 1849), *Pilophorus confusus* (Kirschbaum, 1856), *Ploiaria chilensis* (Philippi, 1862), *Sophonia orientalis* (Matsumura, 1912) and *Textrix caudata* L. Koch, 1872.

Calculation of the Biotic Integrity Index (IBI) showed that SMR-SNAILS-T04 consistently recorded high values, whereas SMR-SNAILS-T08 exhibited the clearest decline over time. In this study, we extended the long-term monitoring of the mixed forests of Santa Maria by deploying SLAM (Sea, Land, Air, Malaise) traps and compiling a comprehensive database of all identified specimens collected between Summer 2024 and Autumn 2025.

## Introduction

Oceanic islands contain highly distinctive biotas, with many endemic species that are vulnerable to habitat loss, biological invasions and other anthropogenic pressures (Triantis et al. 2010, Tershy et al. 2015, Whittaker et al. 2017). Terrestrial invertebrates play fundamental roles in ecosystem functioning and contribute to several ecosystem services (Allan et al. 2015, Bennett et al. 2015, Noriega et al. 2018), such as biological control (Altieri 2018), seed dispersion (Henao-Gallego et al. 2012), nutrient cycling (McCary and Schmitz 2021) and pollination (Martins and Antonini 2016), all of which are crucial and contribute, directly or indirectly, to human health and well-being (Sandifer et al. 2015).

The Azores Archipelago has undergone substantial anthropogenic transformation since Portuguese colonisation in the 15^th^ century, including urban development, agricultural expansion and exploitation of native forests, resulting in the introduction of invasive and exotic species, forest fragmentation, declining native vegetation and endemic biodiversity (Gaspar et al. 2008, Triantis et al. 2010, Fernández-Palacios et al. 2011, Gaspar et al. 2011). As a result, pristine native forest now occupies only a very small proportion of the Archipelago and remaining fragments are often threatened by invasive alien plants and associated habitat degradation (Gaspar et al. 2008, Elias et al. 2016, Borges et al. 2019, Tsafack et al. 2023b).

Arthropods are widely recognised as sensitive indicators of habitat quality (Borges et al. 2021) because they can respond rapidly to climatic variation (Wallon et al. 2024), environmental disturbance (Koivula 2011) and habitat degradation (Meijer et al. 2010). Endemic species, in particular, are often restricted to native forest, as demonstrated in various studies (Ribeiro et al. 2005, Borges et al. 2017, Borges et al. 2020). A study conducted in Santa Maria Island showed that distance from native forest was negatively correlated with the occurrence of single-island endemic species (Meijer et al. 2010). In contrast, introduced species tend to be more abundant in exotic and disturbed forest (Cardoso et al. 2007, Cardoso et al. 2009, Meijer et al. 2010, Tsafack et al. 2021, Tsafack et al. 2023a). These changes are especially relevant for arthropods, because Azorean endemic species are often associated with native forest habitats, whereas introduced species tend to be more frequent in disturbed, exotic or human-modified habitats (Ribeiro et al. 2005, Cardoso et al. 2009, Meijer et al. 2010, Borges et al. 2020). Recent Azorean studies further show that arthropod communities provide relevant information on long-term biodiversity change, introduced-species dynamics and conservation status in island forests (Borges et al. 2020, Lhoumeau and Borges 2023, Oyarzabal et al. 2024).

Biotic integrity indices provide a practical way to summarise complex community information into indicators that can support conservation management (Cardoso et al. 2007, Gudiño-Sosa et al. 2023, Tsafack et al. 2023a, Tsafack et al. 2023b). Although terrestrial ecosystems are the focus of extensive research and conservation efforts, the majority of biotic integrity indices have been developed for freshwater and marine environments rather than for terrestrial systems (Katsiapi et al. 2016, Zhu et al. 2021). In the Azores, arthropod-based Index of Biotic Integrity approaches were first developed for epigean communities and later extended to SLAM trap and canopy communities, allowing habitat quality to be assessed across different forest strata (Cardoso et al. 2007, Tsafack et al. 2023a, Tsafack et al. 2023b). The IBI-SLAM index is particularly relevant for long-term monitoring because it uses arthropods collected with Sea, Land and Air Malaise traps and combines seven metrics related to endemic, native, saprophagous and introduced components of the community, producing a score from 0 to 14, where higher values indicate higher biotic integrity (Tsafack et al. 2023a, Tsafack et al. 2023b). As IBI-SLAM values vary seasonally, temporal comparisons should account for sampling season, particularly when assessing conservation interventions or changes in habitat quality through time (Tsafack et al. 2023b).

The LIFE SNAILS (Support and Naturalisation in Areas of Importance for Land Snails) project aims to protect two endemic terrestrial snails of Santa Maria, *Leptaxis minor* Backhuys, 1975, *Oxychilus agostinhoi* Martins, 1981, endemic semi-slug *Azorivitrina angulosa* (Morelet, 1860) and their habitats. *Azorivitrina angulosa* and *Oxychilus agostinhoi* are critically endangered and *Leptaxis minor* is endangered, based on the IUCN Red List assessments (Cameron et al. 2020, de Frias Martins and Teixeira 2025a, de Frias Martins and Teixeira 2025b). Although arthropods are not the direct conservation targets of LIFE SNAILS, they provide an operational proxy for the ecological condition of the habitats that support threatened endemic invertebrates on Santa Maria Island (Cardoso et al. 2007, Tsafack et al. 2023a, Tsafack et al. 2023b, Borges et al. 2024).

## General description

### Purpose

The first LIFE-SNAILS arthropod data paper established a baseline for Santa Maria Island using 11 SLAM traps monitored from September to December 2022 in mixed forests, recording 118 arthropod taxa and providing initial IBI values for the sampled sites (Borges et al. 2024). The present contribution extends that baseline with new monitoring data from Santa Maria Island and applies the IBI-SLAM framework to evaluate habitat quality under the scope of the LIFE-SNAILS project (Tsafack et al. 2023a, Tsafack et al. 2023b, Borges et al. 2024). Specifically, this study aims to: (i) provide an updated inventory of arthropods sampled with SLAM traps in LIFE-SNAILS intervention areas; (ii) assess spatial and temporal variation in IBI-SLAM values amongst monitored sites; and (iii) support the evaluation of habitat quality for threatened endemic invertebrates in Santa Maria Island through standardised, repeatable arthropod monitoring.

## Project description

### Title

The use of arthropods as surrogates of habitat quality within the scope of LIFE SNAILS project.

### Personnel

The SLAM monitoring protocol was conceived and led by Paulo A.V. Borges.

Fieldwork (site selection and experimental setting): António Manuel de Frias Martins, Mauro Ponte, Nelson B. Moura, Paulo A.V. Borges and Ricardo J.F. Abreu.

Fieldwork (authorisation): Secretaria Regional do Ambiente e Ação Climática (Licenças 23/2024/DRAAC 106/2025/DRAAC) and Direção Regional da Ciência, Inovação e Desenvolvimento (CCIR-RAA/2024/7; CCIR-RAA/2025/54).

Fieldwork: Nelson B. Moura and Paulo A. V. Borges.

Parataxonomists: Alejandro Torres Expósito, Alexandra Dal Lago, Ambre Solivellas, Amelie Neitzel, Cas Beekhuizen, Guillaume Péron, José Luis Laguna Fernández, Luna Serrano Navarro, Martha Vounatsi, Noelia Reverón García, Sophie Wallon, Vid de Graaf and Yeray Gómez López.

Taxonomist: Paulo A. V. Borges.

Voucher specimen management: Alexandra Dal Lago, Martha Vounatsi and Sophie Wallon.

Database management and Darwin Core databases: Paulo A. V. Borges and Sébastien Lhoumeau.

### Study area description

Santa Maria is a small volcanic island located in the eastern group of the Azores Archipelago (together with São Miguel), covering an area of 97.2 km^2^ and reaching a maximum elevation of 590 m a.s.l. The Island exhibits a temperate oceanic climate, associated with frequent and substantial rainfall, consistently high relative humidity and persistent winds, particularly during autumn and winter. The sampling areas are characterised mostly by mixed forests composed of endemic, native and introduced plant species. Amongst the dominant native and endemic flora are *Morella faya*, *Erica azorica*, *Picconia azorica*, *Vaccinium cylindraceum* and *Laurus azorica*. Exotic vegetation is represented by species such as *Pittosporum undulatum* and *Hedychium gardnerianum*, as well as forestry plantations of *Cryptomeria japonica*.

### Funding

Secretaria Regional do Ambiente e Alterações Climáticas, Project LIFE SNAILS (LIFE20 NAT/PT/001377) and “Upgrading the Azorean Biodiversity Portal Infrastructure (AZORES BIOPORTAL- PORBIOTA) to Boost Biodiversity Research, Management and Education -PORBIOTA” (DRCID, ACORES2030-FEDER-03420600).

EU ERASMUS+ Training Grants to Alejandro Torres Expósito, Alexandra Dal Lago, Ambre Solivellas, Amelie Neitzel, Cas Beekhuizen, Guillaume Péron, José Luis Laguna Fernández, Luna Serrano Navarro, Noelia Reverón García, Vid de Graaf and Yeray Gómez López.

## Sampling methods

### Study extent

**Figure 1.**
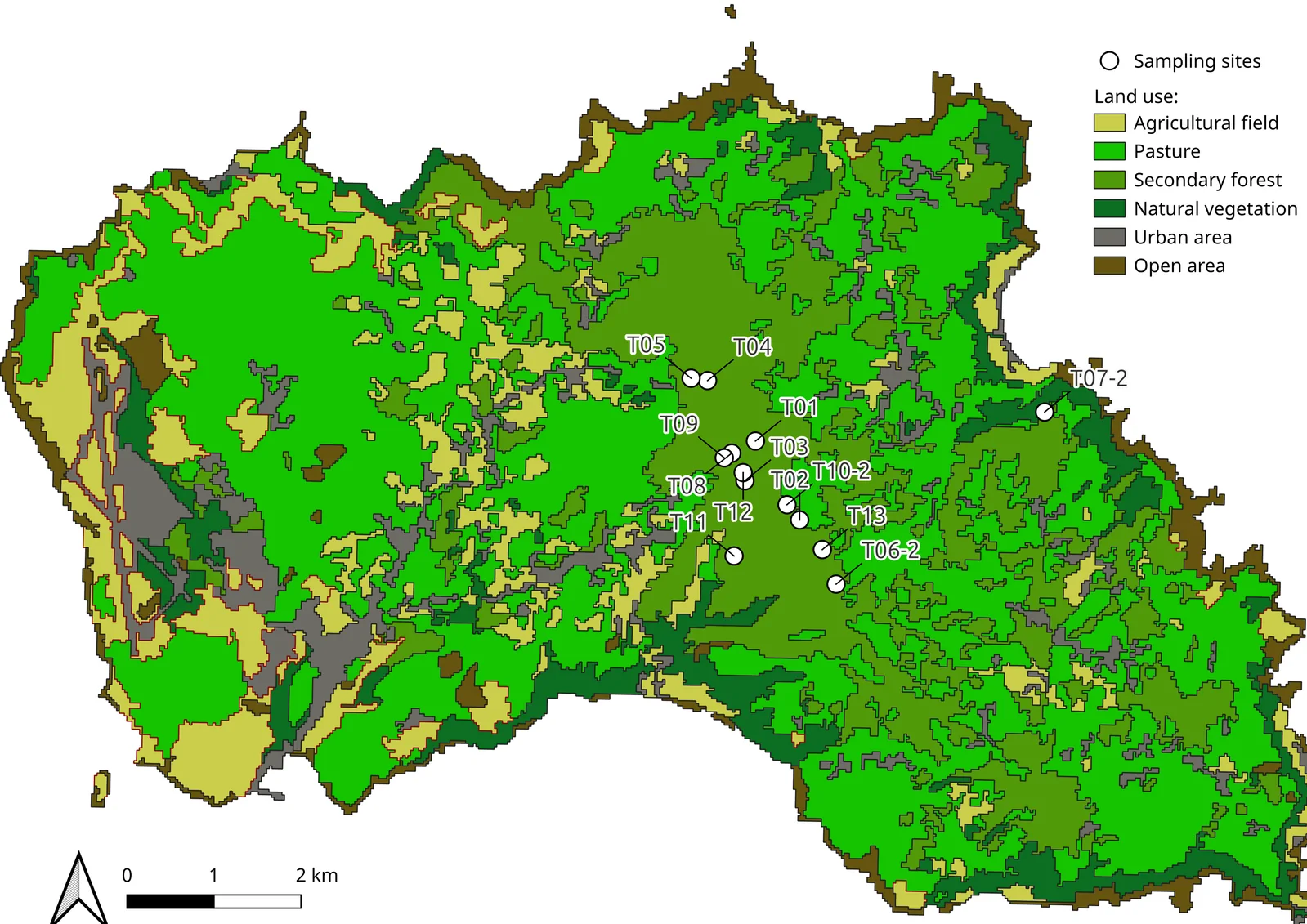
Santa Maria Island with the locations of the study sites. For details on the sites, see Tables 1, 2.

Eleven sites were sampled throughout most of the study period, with two additional sites included during the final sampling season (Table 1; Fig. 1). The sampling sites consisted mainly of mixed forests containing endemic, native non-endemic and introduced plant species. The predominant native and endemic species include *Morella faya, Erica azorica, Picconia azorica, Vaccinium cylindraceum* and *Laurus azorica*. Introduced vegetation is represented by species such as P*ittosporum undulatum* and *Hedychium gardnerianum*, together with forestry plantations of *Cryptomeria japonica*. Information on vegetation composition, particularly the dominant plant species in the surrounding area, was also recorded (Table 2).

### Sampling description

**Figure 2.**
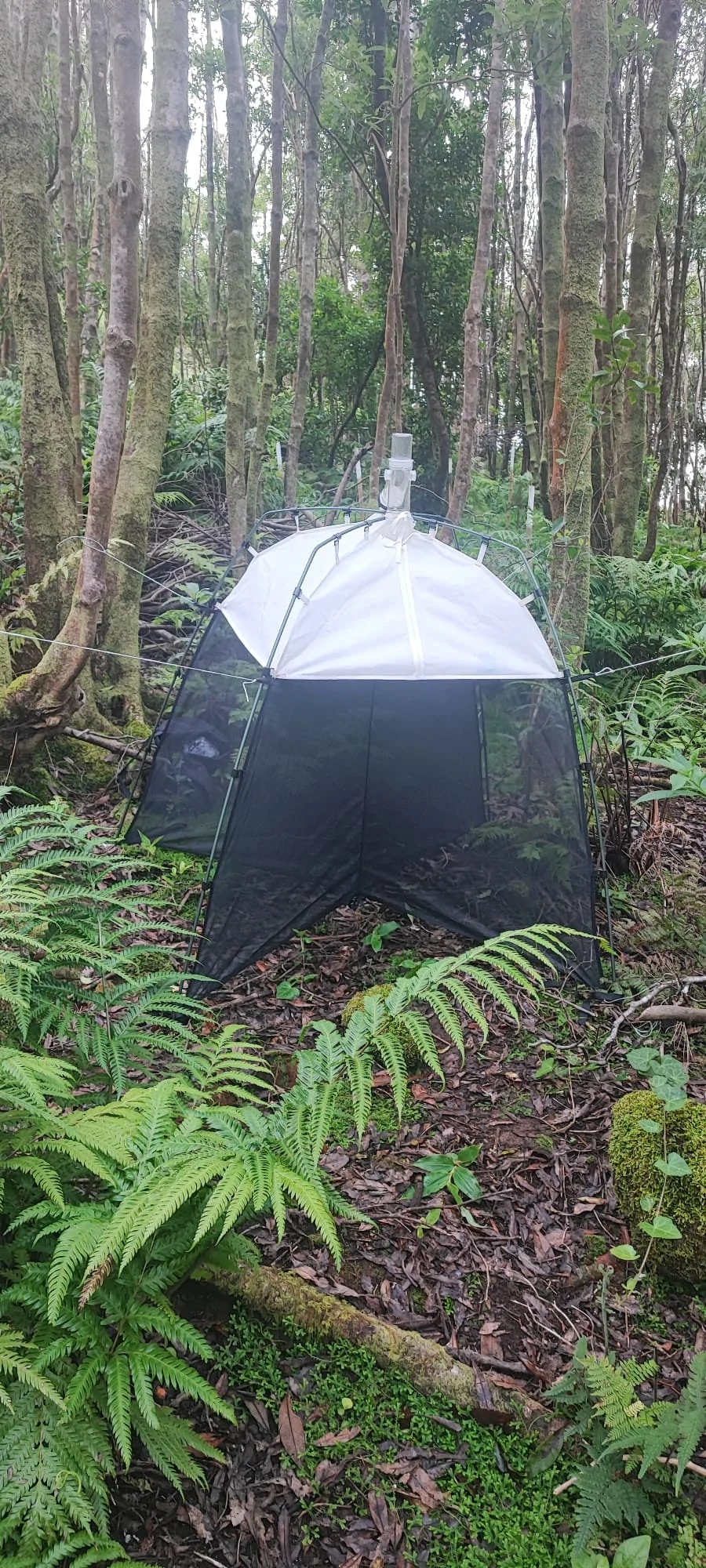
SLAM traps (Sea, Land and Air Malaise) in the new site SMR-SNAILS-T12 (Credit: Paulo A. V. Borges).

We deployed passive flight-interception SLAM traps (Sea, Land and Air Malaise) (Fig. 2) at 13 sites of Santa Maria Island, most of which were characterised by mixed forest vegetation. Eleven sites were monitored throughout the survey period. Two additional sites were included in the final sampling season. The trap is composed of a 110 cm x 110 cm x 110 cm structure that guides arthropods upwards along a mesh surface, after which they drop into a collecting container positioned inside the device (Borges et al. 2017). Each container is filled with propylene glycol (pure 1,2-propanediol), which both kills the captured arthropods and preserves the specimens. The trap collects flying insects, but also non-flying species as it functions as an artificial extension of the tree, allowing organisms such as spiders to climb into it as well (Borges et al. 2017). As a result, this technique broadens the diversity of arthropod groups sampled.

### Quality control

Species nomenclature and colonisation status were assigned according to the most recent checklist of Azorean arthropods (Borges et al. 2022). Preservation in ethanol and appropriate storage of the specimen collection was ensured by Martha Vounatsi.

### Step description

Samples were collected every sampling season, specimens being sorted by parataxonomists and identified by taxonomist Paulo A. V. Borges, following standard laboratory procedures. Based on morphological and anatomical characters, including reproductive structures, specimens were assigned to a morphospecies code number and their respective scientific species or subspecies name (when identification at the level of species or subspecies was possible).

In the Supplementary Material (Suppl. material 1), it is explained how to calculate and interpret the IBI-SLAM, an arthropod-based Index of Biotic Integrity designed to assess the ecological condition of Azorean forest sites using assemblages collected with Sea, Land and Air Malaise traps (SLAM traps) (Fig. 2) (Tsafack et al. 2023b).

The IBI-SLAM is part of a family of Azorean arthropod-based biotic integrity indices that also includes an epigeal index, based on pitfall-trap communities (Cardoso et al. 2007) and a canopy index, based on canopy-beating communities (Tsafack et al. 2023b).

The index was developed for Azorean forest arthropod communities and is intended for comparative monitoring of site condition, especially in conservation and restoration contexts where native forest quality, endemic species persistence and introduced species pressure must be assessed in an integrated way (Tsafack et al. 2023a, Tsafack et al. 2023b).

As the index was calibrated using Azorean arthropod assemblages, it should not be transferred uncritically to other regions or habitats, although its multi-metric logic can provide information for similar island-based monitoring tools when endemic, native non-endemic and introduced taxa can be classified consistently (Tsafack et al. 2023b).

## Geographic coverage

### Description

This survey is focused on the island of Santa Maria (Azores, Portugal).

### Coordinates

36°57'47.76'' and 36°59'4.27'' Latitude; -25°3'0.8'' and -25°5'45.11'' Longitude.

## Taxonomic coverage

### Description

Kingdom: Animalia.

Phylum: Arthropoda.

Class: Arachnida, Chilopoda, Diplopoda, Insecta.

Order: Araneae, Archaeognatha, Blattodea, Chordeumatida, Coleoptera, Dermaptera, Diptera, Geophilomorpha, Hemiptera, Hymenoptera, Julida, Lithobiomorpha, Neuroptera, Opiliones, Orthoptera, Phasmida, Polydesmida, Pseudoscorpiones, Psocodea, Thysanoptera

## Traits coverage

Traits of some of the species can be found in Oyarzabal et al. (2026).

## Temporal coverage

**Data range:** 2024-6-16 – 2025-12-15.

## Collection data

### Collection name

Dalberto Teixeira Pombo insect collection at the University of Azores.

### Collection identifier

DTP

### Parent collection identifier

DTP

### Specimen preservation method

All specimens were preserved in 96% ethanol. 

### Curatorial unit

Dalberto Teixeira Pombo insect collection at the University of the Azores (Curator: Paulo A. V. Borges).

## Usage licence

### Usage licence

Creative Commons Public Domain Waiver (CC-Zero)

## Data resources

### Data package title

Monitoring arthropods under the scope of LIFE SNAILS project -Part II

### Resource link

https://doi.org/10.15468/qkk7ew

### Alternative identifiers

https://ipt.gbif.pt/ipt/resource?r=life_snails_part2&v=1.0

### Number of data sets

2

### Data set 1.

#### Data set name

Event Table

#### Data format

Darwin Core Archive

#### Character set

UTF-8

#### Download URL

https://ipt.gbif.pt/ipt/resource?r=life_snails_part2

#### Data format version

1.1

#### Description

The dataset was published in the Global Biodiversity Information Facility platform (GBIF, Vounatsi et al. 2026). The dataset includes records for which a taxonomic identification of the species was possible. The dataset is structured as a sampling event dataset and published through GBIF as a Darwin Core Archive (DwCA), which is a standardised format for sharing biodiversity data as data tables. The core data file contains 55 records (eventID). The GBIF IPT archives the data and serves as a data repository where the data and resource metadata are available for download.

**Data set 1.** 

Column label	Column description
eventID	Identifier of the events, unique for the dataset.
stateProvince	Name of the region of the sampling site (Azores).
islandGroup	Name of the archipelago (Azores).
island	Name of the island (Santa Maria).
country	Country of the sampling site (Portugal).
countryCode	ISO code of the country of the sampling site (PT).
municipality	Municipality of the sampling sites (Vila do Porto).
minimumElevationInMetres	The lower limit of the range of elevation (altitude, above sea level), in metres.
decimalLongitude	Approximate centre point decimal longitude of the field site in GPS coordinates.
decimalLatitude	Approximate centre point decimal latitude of the field site in GPS coordinates.
geodeticDatum	The ellipsoid, geodetic datum or spatial reference system (SRS), upon which the geographic coordinates given in decimalLatitude and decimalLongitude are based.
coordinateUncertaintyInMetres	Uncertainty of the coordinates of the centre of the sampling plot.
coordinatePrecision	Precision of the coordinates.
georeferenceSources	A list (concatenated and separated) of maps, gazetteers or other resources used to georeference the Location, described specifically enough to allow anyone in the future to use the same resources.
locationID	Identifier of the location.
locality	Name of the locality.
habitat	The habitat of the sample.
year	Year of the event.
eventDate	Date or date range the record was collected.
sampleSizeValue	The numeric amount of time spent in each sampling.
sampleSizeUnit	The unit of the sample size value.
eventRemarks	Information is given on the sampling season
samplingProtocol	The sampling protocol used to capture the species (SLAM traps).

### Data set 2.

#### Data set name

Occurrence Table

#### Data format

Darwin Core Archive

#### Character set

UTF-8

#### Download URL

https://ipt.gbif.pt/ipt/resource?r=life_snails_part2

#### Data format version

1.1

#### Description

The dataset was published through the Global Biodiversity Information Facility platform, GBIF (Vounatsi et al. 2026). The following data table includes all the records for which a taxonomic identification of the species was possible. The dataset submitted to GBIF is structured as an occurrence table that has been published as a Darwin Core Archive (DwCA), which is a standard format for sharing biodiversity data as a set of one or more data tables. The core data file contains 1928 records (occurrenceID). This GBIF IPT archives the data and serves as the data repository. The data and resource metadata are available for download in the Portuguese GBIF Portal IPT.

**Data set 2.** 

Column label	Column description
eventID	Identifier of the events, unique for the dataset.
type	Type of the record, as defined by the Public Core standard.
licence	Reference to the licence under which the record is published.
institutionID	The identity of the institution publishing the data.
collectionID	The identity of the collection publishing the data.
collectionCode	The code of the collection where the specimens are conserved.
institutionCode	The code of the institution publishing the data.
DatasetName	Name of the dataset.
basisOfRecord	The nature of the data record.
recordedBy	A list (concatenated and separated) of names of people, groups or organisations who performed the sampling in the field.
occurrenceID	Identifier of the record, coded as a global unique identifier.
organismQuantity	A number or enumeration value for the quantity of organisms.
organismQuantityType	The type of quantification system used for the quantity of organisms.
sex	The sex and quantity of the individuals captured.
lifeStage	The life stage of the organisms captured.
establishmentMeans	The process of establishment of the species in the location, using a controlled vocabulary: 'native', 'introduced', 'endemic', "uncertain".
identifiedBy	A list (concatenated and separated) of names of people, groups or organisations who assigned the Taxon to the subject.
dateIdentified	The date on which the subject was determined as representing the Taxon.
scientificName	Complete scientific name including author and year.
kingdom	Kingdom name.
phylum	Phylum name.
class	Class name.
order	Order name.
family	Family name.
genus	Genus name.
specificEpithet	Specific epithet.
infraspecificEpithet	Infraspecific epithet.
taxonRank	Lowest taxonomic rank of the record.
scientificnameAuthorship	Name of the author of the lowest taxon rank included in the record.
identificationRemarks	Information about morphospecies identification (code in Dalberto Teixeira Pombo Collection).
occurrenceStatus	A statement about the presence or absence of a dwc:Taxon at a dcterms:Location.

## Additional information

Overall, 20,282 specimens were collected, of which 15,055 were identified to species or subspecies level, corresponding to 74% of the total sample. In total, 172 species and subspecies were recorded, comprising 27 endemic, 48 native non-endemic, 77 introduced and 20 taxa of uncertain colonisation status. A total of 4,865 individual specimens were endemic, 6,075 were native non-endemic and 3,881 were introduced. Notably, the endemic species *Cixius azomariae* Remane & Asche, 1979 (Hemiptera-Cixiidae; n = 1825), *Elipsocus azoricus* Meinander, 1975 (Psocodea-Elipsocidae; n = 695) and *Rugathodes acoreensis* Wunderlich, 1992 (Araneae-Theridiidae; n = 600) were particularly abundant.

A total of ten new island records were documented for Santa Maria Island (Table 3), comprising one introduced spider (Araneae), one introduced pseudoscorpion (Pseudoscorpiones), three beetles (Coleoptera), four hemipterans, including two native non-endemic species and one introduced dipteran. The chrysomelid *Phyllotreta procera* (Redtenbacher, 1849) was first documented in the Azores, on Pico Island, in 2019 (see MF1709 in Borges and Lhoumeau (2024)), although the species had previously been collected on Graciosa Island in 2015, 2016 and 2020 (Borges et al. 2026). It was also found in São Miguel Island in 2025 (Leite et al. 2026). In the present study, a single individual was recorded in Autumn 2025, at site T09 which is dominated by introduced vegetation. Both in São Miguel and Santa Maria, only one individual of the species was found (Leite et al. 2026). Although not yet abundant, the species appears to be gradually spreading across the Azores Archipelago, mirroring its expansion observed in other parts of Europe (Molander and Hellqvist 2011).

*Phyllotreta procera* occurs across Europe, Central Asia, eastern Africa as well as in Macaronesia (Cabo Verde, Canary Islands and Madeira) (Erber 1986, Romantsov and Rakhimov 2023). Although some species of the genus can damage agricultural crops, the known host plants of *P. procera* belong to the Resedaceae and no species-specific crop-damage has yet been documented (Dedyukhin 2014, Bieńkowski and Orlova-Bienkowskaja 2015).

*Hermetia illucens* is a polysaprophagous species native of the Neotropical Region that has expanded across many warm regions of the world (Marshall et al. 2015). It is widely bred as it has numerous applications and can be useful as bioremediator, more specifically entomoremediator (Bulak et al. 2018). Its byproducts can be used as animal and fish food in agriculture and aquaculture, respectively, as a good source of protein and fats (Smetana et al. 2019, Mohan et al. 2022), biofuel (Li et al. 2011), even a biopolymer that can be used in medicine due to its antimicrobial and antifungal properties (Guarnieri et al. 2022). *Hermetia illucens* (Linnaeus, 1758), commonly known as black soldier fly, was first recorded in the Azores on São Miguel Island and more specifically, in two sites (Boieiro and Borges 2024). In the present survey, only one individual was recorded.

According to the Index of Biotic Integrity (IBI) framework (see for the explanation and rationale of the IBI the Suppl. material 1), proposed by Cardoso et al. (2007) and Tsafack et al. (2023b), the IBI values calculated for each sampling site are presented in Table 4. Lower IBI scores were mainly associated with factors linked to introduced species, which tend to be more common in forests dominated by exotic vegetation and are generally more tolerant of environmental disturbance (Cardoso et al. 2007, Tsafack et al. 2021, Tsafack et al. 2023a, Tsafack et al. 2023b).

According to the adopted scoring thresholds, IBI-SLAM values predominantly fell within the moderate condition category across the monitored LIFE SNAILS sites; no observations were classified as poor and only a few reached the good category (Tsafack et al. 2023b). Mean values increased slightly from Summer 2024 to Winter–Spring 2025 and subsequently declined in Summer–Autumn 2025, indicating temporal variation rather than a consistent directional trend (Tsafack et al. 2023a). Amongst the sites, SMR-SNAILS-T04 displayed the most consistently high IBI-SLAM values, whereas SMR-SNAILS-T08 showed the clearest decrease over the sampling period. By contrast, SMR-SNAILS-T09 and SMR-SNAILS-T11 showed moderate increases during the later sampling periods. Overall, these results indicate relatively stable, but spatially heterogeneous IBI-SLAM scores. As the index integrates several community attributes, including metrics based on the relative representation of endemic, native non-endemic and introduced arthropods, variation in these components may have contributed to the observed patterns (Tsafack et al. 2023a, Tsafack et al. 2023b). However, these potential relationships were not tested independently; consequently, differences in IBI-SLAM values should not be interpreted as direct evidence of differences or changes in habitat quality.

## Supplementary Material

814322D4-D900-5AAD-9714-21108D5AF8E4 10.3897/BDJ.14.e203118.suppl1 0 Supplementary material 1 Index of Biotic Integrity based on SLAM-trap arthropod communities (IBI-SLAM): rationale, parameters, scoring and interpretation Data type PDF Brief description This Supplementary Material explains how to calculate and interpret the IBI-SLAM, an arthropod-based Index of Biotic Integrity designed to assess the ecological condition of Azorean forest sites using assemblages collected with Sea, Land and Air Malaise traps (SLAM traps). File: oo_1767157.pdf https://binary.pensoft.net/file/1767157 Borges, P.A.V.

## Figures and Tables

**Table 1.** Details on sites with the decimal longitude and latitude and minimum elevation in metres (m).

Site Code	Locality	Longitude	Latitude	Elevation (m)
SMR-NFPA-T01	Pico Alto T01	-25.087560	36.978040	460
SMR-SNAILS-T02	Casa dos Picos	-25.081848	36.969886	400
SMR-SNAILS-T03	Linha Média Tensão	-25.088924	36.973938	374
SMR-SNAILS-T04	Trilho BTT_Alto Nascente_1	-25.093743	36.984282	443
SMR-SNAILS-T05	Trilho BTT_Alto Nascente_2	-25.095865	36.984520	400
SMR-SNAILS-T06-2	Fontinhas Florestal Miradouro	-25.077136	36.963267	400
SMR-SNAILS-T07-2	Ribeira do Salto	-25.050221	36.981027	208
SMR-SNAILS-T08	Trilho Areia Branca 1	-25.090618	36.976793	419
SMR-SNAILS-T09	Trilho Areia Branca 2	-25.091595	36.976312	397
SMR-SNAILS-T10-2	Piquinhos	-25.083498	36.971465	423
SMR-SNAILS-T11	Ribeira da Fonte Rainha	-25.090315	36.966181	188
SMR-SNAILS-T12	Talhão B9	-25.08915	36.974789	377
SMR-SNAILS-T13	Talhão G Fontinhas	-25.078916	36.966839	431

**Table 2.** Details on the plant species in each site.

Site Code	Locality	Main Plant Composition
SMR-NFPA-T01	Pico Alto T01	Endemic plants: *Picconia azorica*, *Erica azorica*, *Laurus azorica*, *Vaccinium cylindraceum*. Native plants: *Morella faya*, *Myrsine retusa*. Exotic invasive plants: *Pittosporum undulatum*, *Hedychium gardnerianum*. High diversity of endemic and native Pteridophyta and Bryophyta.
SMR-SNAILS-T02	Casa dos Picos	Exotic invasive plants: *Pittosporum undulatum*, *Hedychium gardnerianum*, *Acacia* spp.
SMR-SNAILS-T03	Linha Média Tensão	Endemic plants: *Picconia azorica*. Exotic invasive plants: *Cryptomeria japonica*, *Pittosporum undulatum*, *Hedychium gardnerianum*, *Rubus ulmifolius*. High diversity of endemic and native Pteridophyta and Bryophyta.
SMR-SNAILS-T04	Trilho BTT_Alto Nascente_1	Endemic plants: *Picconia azorica*. Exotic invasive plants: *Cryptomeria japonica*, *Pittosporum undulatum*, *Hedychium gardnerianum*, *Pisidium* sp. High diversity of endemic and native Pteridophyta and Bryophyta.
SMR-SNAILS-T05	Trilho BTT_Alto Nascente_2	Exotic invasive plants: *Eucalyptus* spp., *Pittosporum undulatum*, *Hedychium gardnerianum*, *Pisidium* sp. High diversity of endemic and native Pteridophyta and Bryophyta.
SMR-SNAILS-T06-2	Fontinhas Florestal Miradouro	Endemic plants: *Picconia azorica*, *Laurus azorica*. Exotic invasive plants: *Cryptomeria japonica*, *Pittosporum undulatum*, *Hedychium gardnerianum*, *Rubus ulmifolius*.
SMR-SNAILS-T07-2	Ribeira do Salto	Endemic plants: *Picconia azorica*. Exotic invasive plants: *Pittosporum undulatum*, *Hedychium gardnerianum*. High diversity of endemic and native Pteridophyta and Bryophyta.
SMR-SNAILS-T08	Trilho Areia Branca 1	Exotic invasive plants: *Pittosporum undulatum*, *Hedychium gardnerianum*. High diversity of endemic and native Pteridophyta and Bryophyta.
SMR-SNAILS-T09	Trilho Areia Branca 2	Exotic invasive plants: *Cryptomeria japonica*, *Hedychium gardnerianum*.
SMR-SNAILS-T10-2	Piquinhos	Endemic plants: *Picconia azorica, Erica azorica*. Exotic invasive plants: *Pittosporum undulatum*, *Hedychium gardnerianum*. High diversity of endemic and native Pteridophyta and Bryophyta.
SMR-SNAILS-T11	Ribeira da Fonte Rainha	Exotic invasive plants: *Pittosporum undulatum, Hedychium gardnerianum*.
SMR-SNAILS-T12	Talhão B9	Endemic plants: *Picconia azorica*. Exotic invasive plants: *Pittosporum undulatum*, *Hedychium gardnerianum*, *Rubus ulmifolius*. High diversity of endemic and native Pterydophyta and Bryophyta.
SMR-SNAILS-T13	Talhão G Fontinhas	Endemic plants: *Picconia azorica*. Exotic invasive plants: *Pittosporum undulatum*, *Hedychium gardnerianum*, *Rubus ulmifolius*. High diversity of endemic and native Pterydophyta and Bryophyta.

**Table 3.** Inventory of species newly recorded from Santa Maria Island between June 2024 and December 2025, in mixed forest sites of Santa Maria Island (Azores), including Class, Order and Family names, colonisation status (CS) (NAT - native non-endemic; INT - introduced species; UNC - uncertain origin) (Borges et al. 2022) and total abundance (N) (including adult and juvenile specimens).

Class	Order	Family	Species	CS	N
Arachnida	Araneae	Agelenidae	*Textrix caudata* L. Koch, 1872	INT	2
Arachnida	Pseudoscorpiones	Neobisiidae	*Neobisium maroccanum* Beier, 1930	INT	1
Insecta	Coleoptera	Staphylinidae	*Carpelimus troglodytes troglodytes* (Erichson, 1840)	UNC	7
Insecta	Coleoptera	Staphylinidae	*Nacaeus impressicollis* (Motschulsky, 1858)	UNC	1
Insecta	Coleoptera	Chrysomelidae	*Phyllotreta procera* (Redtenbacher, 1849)	INT	1
Insecta	Diptera	Stratiomyidae	*Hermetia illucens* (Linnaeus, 1758)	INT	1
Insecta	Hemiptera	Cicadellidae	*Sophonia orientalis* (Matsumura, 1912)	INT	4
Insecta	Hemiptera	Miridae	*Campyloneura virgula* (Herrich-Schaeffer, 1835)	NAT	27
Insecta	Hemiptera	Miridae	*Pilophorus confusus* (Kirschbaum, 1856)	NAT	2
Insecta	Hemiptera	Reduviidae	*Ploiaria chilensis* (Philippi, 1862)	INT	1

**Table 4.** Index of Biotic Integrity (IBI) values on a 0 to 14 scale points for each sampling site and season, from Summer 2024 to Autumn 2025.

Site ID	Summer 2024	Winter 2025	Spring 2025	Summer 2025	Autumn 2025
SMR-NFPA-T01	8	10	10	9	8
SMR-SNAILS-T02	9	10	8	10	7
SMR-SNAILS-T03	9	10	9	8	7
SMR-SNAILS-T04	10	10	9	11	11
SMR-SNAILS-T05	8	7	10	8	7
SMR-SNAILS-T06-2	-	8	8	10	7
SMR-SNAILS-T07-2	7	-	10	7	7
SMR-SNAILS-T08	11	7	8	7	8
SMR-SNAILS-T09	6	7	10	8	8
SMR-SNAILS-T10-2	7	9	9	6	7
SMR-SNAILS-T11	8	10	7	8	10
SMR-SNAILS-T12					9
SMR-SNAILS-T13					8
