# Supplementary material for: Monitoring arthropods under the scope of LIFE-SNAILS project: II - Santa Maria Island data with implementation of the Index of Biotic Integrity
Source: Biodivers Data J. 2026 Sep 17;14:e203118. doi: 10.3897/BDJ.14.e203118 (PMC13613079; doi:10.3897/BDJ.14.e203118)
Supplement: Supplementary file 1 [file bdj-14-e203118-s001.pdf]

Supplementary Material S1

Index of Biotic Integrity based on SLAM-trap arthropod communities (IBI-SLAM): rationale, parameters, scoring and interpretation

1. Purpose and scope

This Supplementary Material explains how to calculate and interpret the IBI-SLAM, an arthropod-based Index of Biotic Integrity designed to assess the ecological condition of Azorean forest sites using assemblages collected with Sea, Land and Air Malaise traps (SLAM traps) (Tsafack et al. 2023b).

The IBI-SLAM is part of a family of Azorean arthropod-based biotic integrity indices that also includes an epigeal index based on pitfall-trap communities (Cardoso et al. 2007) and a canopy index based on canopy-beating communities (Tsafack et al. 2023b).

The index was developed for Azorean forest arthropod communities and is intended for comparative monitoring of site condition, especially in conservation and restoration contexts where native forest quality, endemic species persistence and introduced species pressure must be assessed in an integrated way (Tsafack et al. 2023b).

Because the index was calibrated using Azorean arthropod assemblages, it should not be transferred uncritically to other regions or habitats, although its multimetric logic can inform similar island-based monitoring tools when endemic, native non-endemic and introduced taxa can be classified consistently (Tsafack et al. 2023b).

2. Conceptual background

The Index of Biotic Integrity approach combines several biological metrics into a single value that summarises the capacity of a site to support a balanced and persistent community (Cardoso et al. 2007; Tsafack et al. 2023a,b).

Multimetric indices are useful because single indicators, such as richness or abundance alone, may fail to represent the complexity of biological assemblages and may be sensitive to sampling effort, taxonomic composition or local dominance patterns (Hortal et al. 2006; Tsafack et al. 2023b).

The original Azorean arthropod IBI was developed for epigeal arthropods sampled with pitfall traps (Cardoso et al. 2007), whereas the IBI-SLAM was developed to represent the intermediate forest stratum sampled by SLAM traps (Tsafack et al. 2023b).

SLAM traps collect a broad spectrum of arthropods, including flying and highly mobile taxa, and may also intercept some ground-associated species that reach the trap structure; this makes their assemblage different from both pitfall and canopy-beating samples (Tsafack et al. 2023a,b; Lhoumeau and Borges 2023).

The IBI-SLAM therefore complements, rather than replaces, other stratum-specific indices, because arthropod assemblages and disturbance responses can vary across the vertical structure of island forests (Ulyshen 2011; Tsafack et al. 2023b).

3. Data required to calculate IBI-SLAM

For each site, sampling event or standardised sampling period, the calculation requires the following information for the taxa included in the analysed assemblage (Tsafack et al. 2023b):

- a species or subspecies name, or the finest reliable taxonomic identification used in the monitoring dataset;
- the number of individuals assigned to each taxon;
- the colonisation status of each taxon: endemic, native non-endemic, introduced or indeterminate;
- the trophic group of each taxon, especially whether it is saprophagous/decomposer;
- a consistent sampling unit, such as one site, one trap, one season or a standardised period, so that values are comparable among samples.

Taxa with indeterminate colonisation status are retained in total richness and abundance when calculating denominators, but they do not contribute to the endemic, native non-endemic or introduced numerators unless a status is assigned (Tsafack et al. 2023b).

4. Metrics included in IBI-SLAM

Tsafack et al. (2023b) screened 16 candidate parameters using generalised linear models to test their ability to discriminate preserved native forest sites from disturbed forest sites, and the final IBI-SLAM retained seven parameters with the strongest discriminatory performance for comparison with the earlier epigeal IBI.

Code	Metric	Definition	Ecological interpretation in IBI-SLAM
**%sEnd**	Endemic species richness	Percentage of species/subspecies in the sample that are endemic to the Azores.	Higher values indicate stronger representation of Azorean endemic taxa and contribute positively to biological integrity.
**%sSap**	Saprophagous species richness	Percentage of species/subspecies assigned to saprophagous/decomposer trophic groups.	Lower values indicate higher integrity in this index because saprophagous assemblages in disturbed Azorean forests are often associated with introduced species.
**%nSap**	Saprophagous abundance	Percentage of individuals belonging to saprophagous/decomposer taxa.	Lower values indicate higher integrity when saprophagous abundance is dominated by disturbance-associated taxa.
**%sNat**	Native non-endemic species richness	Percentage of species/subspecies that are native to the Azores but not endemic.	Higher values indicate a richer indigenous component in the SLAM-trap assemblage.
**%nEnd**	Endemic abundance	Percentage of individuals belonging to endemic Azorean taxa.	Higher values indicate stronger dominance of endemic taxa and contribute positively to integrity.
**%nInt**	Introduced abundance	Percentage of individuals belonging to introduced taxa.	Lower values indicate lower introduced-species pressure and higher integrity.
**%nNat**	Native non-endemic abundance	Percentage of individuals belonging to native non-endemic taxa.	Lower values receive higher scores in IBI-SLAM because high native non-endemic abundance was associated with disturbed sites during calibration.

Table S1. Metrics retained for the IBI-SLAM and their interpretation. The selected metrics follow the final parameter set presented by Tsafack et al. (2023b).

5. Calculation of percentage metrics

The IBI-SLAM uses percentages so that sites with different total richness or abundance can be compared more consistently, provided that the sampling design and taxonomic treatment are comparable (Cardoso et al. 2007; Tsafack et al. 2023b).

Metric	Formula	Definitions
**%sEnd**	100 × S_endemic / S_total	S = number of species/subspecies in the specified category; S_total = total species/subspecies richness in the sample.
**%sSap**	100 × S_saprophagous / S_total	S = number of species/subspecies in the specified category; S_total = total species/subspecies richness in the sample.
**%nSap**	100 × N_saprophagous / N_total	N = number of individuals in the specified category; N_total = total abundance in the sample.
**%sNat**	100 × S_native non-endemic / S_total	S = number of species/subspecies in the specified category; S_total = total species/subspecies richness in the sample.
**%nEnd**	100 × N_endemic / N_total	N = number of individuals in the specified category; N_total = total abundance in the sample.
**%nInt**	100 × N_introduced / N_total	N = number of individuals in the specified category; N_total = total abundance in the sample.
**%nNat**	100 × N_native non-endemic / N_total	N = number of individuals in the specified category; N_total = total abundance in the sample.

Table S2. Percentage metrics used to calculate IBI-SLAM. These formulae translate the parameter names used by Tsafack et al. (2023) into reproducible calculations for a data paper.

6. Scoring thresholds and final index value

Each IBI-SLAM metric is converted to a discrete score of 0, 1 or 2 according to the threshold values calibrated by Tsafack et al. (2023b).

A score of 0 indicates a value characteristic of disturbed forest conditions, a score of 1 indicates an intermediate condition, and a score of 2 indicates a value characteristic of better preserved native forest conditions (Cardoso et al. 2007; Tsafack et al. 2023b).

Parameter	Score 0	Score 1	Score 2
**%sEnd**	< 18	18-30	> 30
**%sSap**	> 25	20-25	< 20
**%nSap**	> 33	14-33	< 14
**%sNat**	< 38	38-46	> 46
**%nEnd**	< 35	35-63	> 63
**%nInt**	> 19	5-19	< 5
**%nNat**	> 47	33-47	< 33

Table S3. Reference values for scoring IBI-SLAM metrics. The threshold values correspond to the scoring table presented by Tsafack et al. (2023) and to the reference table provided in the starting document for this Supplementary Material.

The total IBI-SLAM value is calculated as the sum of the seven metric scores:

IBI-SLAM = score(%sEnd) + score(%sSap) + score(%nSap) + score(%sNat) + score(%nEnd) + score(%nInt) + score(%nNat)

The minimum possible value is 0 and the maximum possible value is 14, because seven metrics are each scored from 0 to 2 (Tsafack et al. 2023).

7. Interpretation of IBI-SLAM values

IBI-SLAM value	Integrity class	Interpretation
**0-4**	Poor biological integrity	Assemblage values are closer to disturbed forest conditions, with low representation of endemic taxa, high introduced or disturbance-associated components, or both.
**5-10**	Moderate biological integrity	Assemblage values are intermediate and may indicate partial habitat quality, mixed disturbance signals or transitional/restoration conditions.
**11-14**	Good biological integrity	Assemblage values are closer to better preserved native forest conditions, with high endemic richness/abundance and low introduced or disturbance-associated abundance.

Table S4. Suggested interpretation classes for IBI-SLAM. These classes follow the thresholds in Tsafack et al. (2023): values below 5 indicate poor biological integrity, values from 5 to 10 indicate moderate status and values above 10 indicate good biological integrity.

8. Conditions associated with the maximum IBI-SLAM score

A site receives the maximum IBI-SLAM value of 14 when all seven metrics receive score 2, which corresponds to the reference condition defined during calibration (Tsafack et al. 2023b).

- endemic species richness (%sEnd) higher than 30%;
- saprophagous/decomposer species richness (%sSap) lower than 20%;
- saprophagous/decomposer abundance (%nSap) lower than 14%;
- native non-endemic species richness (%sNat) higher than 46%;
- endemic abundance (%nEnd) higher than 63%;
- introduced abundance (%nInt) lower than 5%;
- native non-endemic abundance (%nNat) lower than 33%.

These conditions should be interpreted together, because the IBI-SLAM is a multimetric index and individual metrics may occasionally give contrasting signals (Cardoso et al. 2007; Tsafack et al. 2023b).

11. Suggested text for the Data Paper

Short version for the Methods or Additional information section:

> *We calculated the Index of Biotic Integrity based on SLAM-trap arthropod communities (IBI-SLAM) following Tsafack et al. (2023). For each sampling unit, we calculated seven percentage metrics: endemic species richness (%sEnd), saprophagous species richness (%sSap), saprophagous abundance (%nSap), native non-endemic species richness (%sNat), endemic abundance (%nEnd), introduced abundance (%nInt) and native non-endemic abundance (%nNat). Each metric was converted to a discrete score of 0, 1 or 2 using the reference thresholds proposed by Tsafack et al. (2023), and the seven scores were summed to obtain an index ranging from 0 to 14. Values*

below 5 were interpreted as poor biological integrity, values from 5 to 10 as moderate integrity and values above 10 as good biological integrity. The IBI-SLAM was interpreted as a community-level proxy of forest biological integrity and habitat quality.

12. Calculation template

Site/Event	S_total	N_total	%sEnd	%sSap	%nSap	%sNat	%nEnd	%nInt	%nNat	IBI-SLAM	Class
Site 1											
Site 2											
Site 3											

Table S6. Empty calculation template that can be filled with site-level or event-level IBI-SLAM values in the data paper or supplementary dataset.

References

Cardoso, P., Borges, P. A. V., & Gaspar, C. (2007). Biotic integrity of the arthropod communities in the natural forests of Azores. Biodiversity and Conservation, 16, 2883-2901. https://doi.org/10.1007/s10531-006-9078-x

Hortal, J., Borges, P. A. V., & Gaspar, C. (2006). Evaluating the performance of species richness estimators: sensitivity to sample grain size. Journal of Animal Ecology, 75, 274-287. https://doi.org/10.1111/j.1365-2656.2006.01048.x

Lhoumeau, S., & Borges, P. A. V. (2023). Assessing the impact of insect decline in islands: exploring the diversity and community patterns of indigenous and non-indigenous arthropods in the Azores native forest over 10 years. Diversity, 15, 753. https://doi.org/10.3390/d15060753

Lhoumeau, S., Tsafack, N., Manso, S., Figueiredo, T., Leite, A., Parmentier, L., Ferreira, M. T., & Borges, P. A. V. (2024). Monitoring arthropods under the scope of the LIFE-BEETLES project: I - Baseline data with implementation of the Index of Biotic Integrity. Biodiversity Data Journal, 12, e124799. https://doi.org/10.3897/BDJ.12.e124799

Stephenson, P. J., Londoño-Murcia, M. C., Borges, P. A. V., et al. (2022). Measuring the impact of conservation: the growing importance of monitoring fauna, flora and funga. Diversity, 14, 824. https://doi.org/10.3390/d14100824

Tsafack, N., Lhoumeau, S., Ros-Prieto, A., Navarro, L., Kocsis, T., Manso, S., Figueiredo, T., Ferreira, M.T. & Borges, P.A.V. (2023a). Biological Integrity of Azorean Native Forests Is better measured in Cold Season. Diversity, 15: 1189; https://doi.org/10.3390/d15121189

Tsafack, N., Lhoumeau, S., Ros-Prieto, A., Navarro, L., Kocsis, T., Manso, S., Figueiredo, T., Ferreira, M. T., & Borges, P. A. V. (2023b). Arthropod-based biotic integrity indices: a novel tool for evaluating the ecological condition of native forests in the Azores archipelago. Ecological Indicators, 154, 110592. https://doi.org/10.1016/j.ecolind.2023.110592

Ulyshen, M. D. (2011). Arthropod vertical stratification in temperate deciduous forests: implications for conservation-oriented management. Forest Ecology and Management, 261, 1479-1489. https://doi.org/10.1016/j.foreco.2011.01.033
